# Mechanochemical preparation of triptolide-loaded self-micelle solid dispersion with enhanced oral bioavailability and improved *anti-tumor* activity

**DOI:** 10.1080/10717544.2022.2069879

**Published:** 2022-05-09

**Authors:** Dabu Zhu, Qiuqin Zhang, Yifang Chen, Minghua Xie, Jianbo Li, Shen Yao, Ming Li, Zhao Lou, Yue Cai, Xuanrong Sun

**Affiliations:** a First People's Hospital of Linping District, Hangzhou, China; b Collaborative Innovation Center of Yangtze River Delta Region Green Pharmaceuticals and College of Pharmaceutical Science, Zhejiang University of Technology, Hangzhou, China

**Keywords:** Triptolide, solid dispersion, nanomicelle, mechanical ball milling, bioavailability, antitumor activity

## Abstract

Triptolide (TP), a compound isolated from a Chinese medicinal herb, possesses potent anti-tumor, immunosuppressive, and anti-inflammatory properties, but was clinically limited due to its poor solubility, bioavailability, and toxicity. Considering the environment-friendly, low-cost mechanochemical techniques and potential dissolution enhancement ability of Na_2_GA, an amorphous solid dispersion (Na_2_GA&TP-BM) consisting of TP and Na_2_GA were well-prepared to address these issues. The performance of Na_2_GA&TP-BM was improved through ball milling, such as from crystalline state to an amorphous solid dispersion, suitable nano micelle size and surface potential, and increased solubility. This change had a significant improvement of pharmacokinetic behavior in mice and could be able to extend the blood circulation time of the antitumor drug. Moreover, *in vitro* and *in vivo* anti-tumor study showed that Na_2_GA&TP-BM displayed more potent cytotoxicity to tumor cells. The work illustrated an environment-friendly and safe preparation of the TP formulation, which was promising to enhance the oral bioavailability and antitumor ability of TP, might be considered for efficient anticancer therapy.

## Introduction

Triptolide, isolated from a Chinese herb called *Tripterygium wilfordii*, has been used in Chinese medicine for centuries to treat inflammatory and autoimmune diseases (Gu et al., 2016). In recent years, triptolide has attracted attention for its ability to inhibit growth and accelerate the death of tumor cells *in vitro* and *in vivo* (L Liu et al., 2018; Jiang et al., 2019; Tian et al., 2019; Deng et al., 2021). Triptolide exhibits stronger antitumor activity than traditional antineoplastic drugs such as doxorubicin, mitomycin, cisplatin, and paclitaxel (Yang et al., 2003). At present, the marketed triptolide is administrated via oral (tablet dosage forms) and intravenous route (parenteral injections). Despite its curative effects on the various diseases, the clinical application of triptolide has been limited due to its poor aqueous solubility, short half-life in the circulation, and serious side effects.

Over the past decades, considerable efforts have been dedicated to designing and developing a variety of TP delivery systems with the intention of improving the drug insolubility, alleviating the adverse toxicity effects and enhancing the bioavailability (Ren et al., 2021), such as solid lipid nanoparticle (Xue et al., 2012; C Zhang et al., 2014; Geszke-Moritz & Moritz, 2016), liposome (Monteiro et al., 2014; Dasa et al., 2015), polymeric micelle (L Xu et al., 2013; Ling et al., 2014), microemulsion (Chen et al., 2004), and so on. In the above methods, a lot of the processes necessitate complicated chemical formulas as well as technically demanding processes. Moreover, various organic solvents (ethanol, dichloromethane, dimethyl sulfoxide, etc.) are usually required. Most of these procedures may increase the risk and the cost of production. These methods involving organic solvents are harmful to human beings and the environment.

Because of the superiority of environment friendliness, easy operation and higher cost performance, more and more attention has been focused on mechanochemistry, such as the development of green synthesis (Bahri et al., 2016; Wei et al., 2018), cocrystal synthesis, and amorphous solid dispersion (W Xu et al., 2018; X Sun et al., 2019). When solid molecular gets high-energy grinding, their particle sizes, crystal forms and physicochemical stability will vary greatly (Boldyrev, 2005). In addition to the above advantages, all the changes may be possible to enhance the solubility and bioavailability (Descamps & Willart, 2016).

Glycyrrhizic acid (GA) is a triterpene glycoside extracted from licorice root, which demonstrates antiviral, anti-inflammatory, and anticancer properties (Pompei et al., 2009; Bernela et al., 2016; Su et al., 2017). Meanwhile, GA forms non-covalent compounds with various drugs due to its amphiphilicity, which significantly increases the solubility of refractory drugs. However, adding GA to water will get a gel-like liquid that is difficult to handle. Na_2_GA, formed by GA and Na ions, solves this problem well. As well as GA, Na_2_GA has the same characteristics because it can undergo hydrolysis in aqueous solutions and form a free GA (Q Zhang et al., 2018). We can expect the synergetic effect of using Na_2_GA as a drug delivery system for TP.

Considering the environment-friendly, low-cost mechanochemical techniques and potential dissolution enhancement ability of Na_2_GA, an amorphous solid dispersion (Na_2_GA&TP-BM) consisting of TP and Na_2_GA were well-prepared. Then, the characterization and behavior of Na_2_GA&TP-BM were studied by using an array of analytical methods such as XRD, SEM; Furthermore, the pharmacokinetics, cytotoxicity studies, and anti-tumor activity *in vitro* and *in vivo* were further investigated.

## Materials and methods

### Materials

TP was obtained from Bide Medical Technology Co., LTD (Shanghai, China, purity >98%). Na_2_GA was supplied by Shanxi Pioneer Biotech Co. Ltd. (Xian, China, purity >98%). Fetal bovine serum (FBS), penicillin/streptomycin (P/S), and Roswell Park Memorial Institute 1640 (RPMI-1640) cell culture medium were all supplied by Gibco BRL (Gaithersburg, MD).

### Cells and animals

The cancer cell lines were all purchased from China Center for Type Culture Collection (Wuhan, China) including the human lung cancer cell line A549, the human hepatocellular carcinoma cell line HepG2, the human colon cancer cell line HCT116, and the human breast cancer cell line MCF-7. HCT116 and A549 were cultured in RPMI-1640 (containing 10% FBS and 1% P/S). MCF-7 and HepG2 cells were cultured in DMEM (containing 10% FBS and 1% P/S). All the cells were routinely maintained in a humidified chamber at 37 °C and 5% CO_2_.

Female ICR mice (5–6 weeks) weighing 18–20 g and BALB/c nude mice (4 weeks) weighing 16–18 g were obtained from the Shanghai Slac Laboratory Animal Co. Ltd. All the animals were performed in strict compliance with the PR China legislation for the use and care of laboratory animals.

### Preparation of solid dispersion by mechanochemical treatment

Ball milling (Planetary Ball Mill PM 400, Retsch) was used to prepare samples. Briefly, accurately weighed 4.500 g Na_2_GA and 0.500 g TP (weight ratio 1/9, named 1/10 SD), or 4.950 g Na_2_GA and 0.050 g TP (weight ratio 1/99, named 1/100 SD), or 4.975 g Na_2_GA and 0.025 g TP (weight ratio 1/199, named 1/200 SD), were added to ball mill pot with 20 steel balls (diameter 12 mm). The grinding time was 30 min, and the rotation speed was 30 rpm. Finally, 1/100 SD was selected for follow-up experiments, described as Na_2_GA&TP-BM. Moreover, a physical mixture consisting of TP and Na_2_GA, described as Na_2_GA&TP-UM, was prepared for comparison with Na_2_GA&TP-BM.

### Solubility determination

Solubility was determined on the samples obtained as follows. Samples were made into a saturated solution and filtered through a filter paper (0.22 µm). The filtrate solution was analyzed by HPLC (Agilent G7129, San Diego, CA) equipped with column Shimadzu ODS-3 C_18_ (Shimadzu, Kyoto, Japan) (4.6 × 250 mm, 5 μm) at 30 °C and diode-array detector was set to a wavelength of 218 nm. The eluent was acetonitrile-deionized water (40:60, v/v), with the flow rate of 1.0 mL/min.

### Dissolution determination

Dissolution tests of pure TP, Na_2_GA&TP-UM and Na_2_GA&TP-BM were performed in a dissolution tester (RC-6ST; Tianjin, China) at the paddle rotation speed of 100 rpm in 900 mL of pH 6.8 phosphate buffer maintained at 37 ± 0.5 °C. Each formulation equivalent to 9 mg of TP was put into dissolution vessel. At the set time point, 2 mL of the sample was extracted, and the phosphoric acid medium was added to the container in time. The samples collected in 1 mL vial were treated with acetonitrile and then filtered into liquid phase vials using a syringe with a filter head. Then, samples were analyzed by HPLC.

### Powder X-ray diffraction (XRD)

The structure of samples was characterized by using X-ray diffract meter (Bruker, Leipzig, Germany). The sampling parameter was the step range of 3°–40° at a speed of 2°/min. Data were processed by Origin 9.0 analysis.

### Scanning electron microscopy (SEM)

A scanning electron microscope (ZEISS Gemini500, Jena, Germany) was used to acquire electronic images. Coating of samples with platinum was performed by a Leica EM ACE200 Vacuum Coater (Germany). The coating parameters were as follows: sputtering time 100 s, amperage 30 mA.

### Polarized light microscopy (PLM)

The crystal characteristics of the samples was observed by polarized light microscopy (Olympus CX41, Tokyo, Japan) and a CCD camera (HTC1600, China). All images were obtained at 10× resolution.

### Molecular dynamics simulations (MD)

The structural optimization based on density functional theory (DFT) was carried out by the Dmol3 module in the Materials Studio (MS) software. The exchange–correlation energy was described with the Perdew Burke Ernzerhof (PBE) version of the generalized gradient approximation (GGA). DFT-D method was employed to calculate the van der Waals (vdW) interaction. The convergence thresholds of the optimized structures were 2.0 × 10^−5 ^Hartree in energy and 0.004 Hartree/Å in force. The maximum number of ionic step and step size were set to 500 and 0.3 Å, respectively.

### Particle characterization and zeta potential

The physicochemical characteristics of samples that dissolved in water were detected by Zetasizer NanoZS (Malvern Instruments, Malvern, UK). Before being measured, all samples were dissolved in deionized water at the concentration of 1 mg/mL. The particle size of the nano-micelle, polydispersity index (PDI), and the charge on its surface (ζ-potential) were detected by dynamic light scattering (DLS) and laser Doppler anemometry.

### Determination of the critical micelle concentration (CMC)

Prepared Nile Red with dichloromethane to make a 1 × 10^−4 ^mol/L solution, and then added to a series of vials. After CH_2_Cl_2_ was evaporated, the aqueous solutions of Na_2_GA&TP-BM with various concentrations ranging from 0.001 to 10 mg/mL were added into the vials, and stirred for 12 h. The fluorescence intensity of these solutions was measured by a microplate reader (Flexstation 3; Molecular Devices LLC, Sunnyvale, CA) at the wavelength of 620 nm (excited at 579 nm) (Ridolfo et al., 2018). Data were processed by Origin 9.0 analysis.

### Transmission electron microscopy (TEM)

The morphology of micelle was observed by using a transmission electron microscope (Hitachi HT700 EXALENS, Tokyo, Japan). Samples were configured into 1 mg/mL solution. One drop of solution was dropped on the surface of the copper sheet and the samples were observed by TEM.

### Multicellular tumor spheroids (MTS) cytotoxicity

MTS of MCF-7 cells were obtained according to the previously reported hanging drop technique (Zhu et al., 2021). MCF-7 cell suspension was diluted in a medium containing 0.24% (w/v) methylcellulose with a density of around 10^5^ cells per milliliter. After 24 h, the spheroids were formed and transferred to agarose-coated 96-well plates with one spheroid in each well. Following attachment, the spheroids were incubated for another 72 h to reach a diameter of approximately 400 µm. Then, Na_2_GA&TP-UM and Na_2_GA&TP-BM at 200 ng/mL TP concentration were added to the 96-well plate every two days and followed by a 2-weeks’ observation. The major (*d*_max_) and minor (*d*_min_) diameters of each spheroid were measured, and the volume of 3D tumor sphere was calculated by using the following formula: *V* = (π × *d*_max_ × *d*_min_)/6 (Ye et al., 2021).

### *In vitro* cytotoxicity study

The MTT assay was used to study the cytotoxicity of TP, Na_2_GA&TP-UM and Na_2_GA&TP-BM on HCT116, A549, MCF-7, and HepG2. Take HepG2 as an example, it was seeded in 96-well plates for 12 h and cultivated in the incubator which had a humidified atmosphere containing 5% CO_2_ at 37 °C. Then, the cells were treated with free TP, Na_2_GA&TP-UM and Na_2_GA&TP-BM (TP concentrations: 0.001–10 µg/mL) for 48 h. Then, 20 µL MTT (5 mg/mL) in PBS was added to each well. After 4 h, the medium was replaced by 200 µL DMSO and shaken at room temperature for 10 min. Finally, the absorbance of each well was detected by using a microplate reader (Flexstation 3; Molecular Devices LLC, Sunnyvale, CA). The operation of other cells was same as above, except the culture medium. The cell viability was calculated according to the following formula: cell viability (%) = cells (samples)/cells (control) × 100 (K Wang et al., 2018).

### Cellular uptake study

In order to observe the cell uptake, Na_2_GA&TP-BM/coumarin-6 (NPs/C6) were prepared as follows. 1 mg Coumarin-6 and 1 mg Na_2_GA&TP-BM were dissolved in 100 µL tetrahydrofuran completely. Then 1 mL deionized water was added dropwise and stirred for extra 2 h. Subsequently, 20 kDa MWCO dialysis bag was used for dialysis of the solution. The labeled NPs/C6 were stored at −20 °C before use.

MCF-7 cells were seeded in 24-well plates at 2 × 10^4^ cells/mL and incubated for 24 h. Next, 1 mL NPs/C6 (concentration: 10 μg/mL) was added to each well. After incubating for 4 h, the cells were washed three times with 4 °C PBS, fixed with 4% paraformaldehyde for 15 min at 25 °C, and stained with 1.5 μg/mL Hoechst for another 10 min. Finally, the plates were observed under a fluorescence microscope (Olympus IX73, Tokyo, Japan).

### Apoptosis study by flow cytometry

Cell apoptosis was detected by Annexin V-FITC/PI staining with flow cytometric analysis (Chou et al., 2010). MCF-7 cells were seeded in 6-well plates and incubated for 12 h. Then, the medium was replaced by 2 mL TP, Na_2_GA&TP-UM, Na_2_GA&TP-BM solution (TP concentrations: 200 ng/mL) and incubated for 24 h. MCF-7 cells were collected after treatment which was performed as the cell cycle experiment. After the cells were washed for three times with PBS, added 100 μL 1× binding buffer (containing 20 μL Annexin V-FITC and 20 μL PI) and incubated for 20 min in the dark at room temperature. Finally, 400 μL 1× binding buffer was added, and the stained cells were tested by flow cytometry.

### Pharmacokinetic evaluation

The pharmacokinetic study of TP, Na_2_GA&TP-UM, and Na_2_GA&TP-BM were performed in ICR mice. Prior to experiments, a total of mice was fasted for 12 h with free access to water, and were randomly divided into three groups (*n* = 5). The dose for each intragastric administration was 0.72 mg/kg (equivalent to TP concentration). After administration 0.083, 0.25, 0.5, 0.75, 1, 2, 8, and 24 h, 0.1 mL of blood samples were collected from the eyelids and placed in the prepared heparinized tube in ice bath, followed by centrifugation at 5000 rpm for 10 min (4 °C) to obtain plasma. All mice remained healthy after blood collection for 8 time points. The plasma samples were prevented from light exposure and flushed with nitrogen gas for storage at −80 °C until analysis. Finally, the samples were detected by HPLC, according to the liquid phase conditions in solubility determination.

### *In vivo* pharmacodynamic evaluation and histological analysis

In order to recognize clearly the therapeutic effects and the side effect to free TP, Na_2_GA&TP-UM, and Na_2_GA&TP-BM on the main organs, *in vivo* anti-tumor therapeutic efficacy evaluations were conducted. First, a subcutaneous HepG2 tumor model of BALB/c nude mice was constructed. After inoculation, nude mice bearing HepG2 cells were randomly divided into four groups (*n* = 6) when the tumor volume reached to 50–80 mm^3^. Then all groups of nude mice were, respectively, given 0.1 mL TP, Na_2_GA&TP-UM, Na_2_GA&TP-BM solution (TP concentrations: 720 μg/kg) and 0.9% saline as control every other day. Throughout the whole treatment process, the body weight, tumor length, and tumor width of mice were recorded before each administration. The tumor volume was measured with a caliper and was calculated as follows: tumor volume = 0.5 × length × width^2^. At the end of the administration period, the mice were humanely sacrificed. Sections of major organs and tumor were fixed in 10% (w/v) neutral-buffered-formalin. After dehydration and embedding, the organs and tumor were sliced and the sections were stained with hematoxylin and eosin (H&E). Finally, the stained sections were observed by using a light microscope.

### Detection of blood biochemical indexes

The blood samples of control, TP, Na_2_GA&TP-UM, Na_2_GA&TP-BM on BALB/c nude mice were collected and centrifuged at 5000 rpm (Beckman Centrifuge D3024, BD Biosciences, Franklin Lakes, NJ) for 5 min to obtain plasma samples. The clinic parameters were measured including heart indices containing creatine kinase (CK), creatine kinase-MB (CK-MB), and lactate dehydrogenase (LDH), liver function-related alanine aminotransferase (ALT) and aspartate aminotransferase (AST), kidney function-associated blood urea nitrogen (BUN) and creatinine (Cr) by an automatic biochemical analyzer (Beckman AU400, BD Biosciences, Franklin Lakes, NJ).

### Statistical analysis

Data were reported as mean ± standard error, using the unpaired Student’s *t*-test. The values of *p < .05, **p < .01, and ***p < .001 calculated by GraphPad Prism 9.2.0 (GraphPad Software, La Jolla, CA) were considered significant and extremely significant, respectively.

## Results and discussion

### Physical characterization studies of TP solid dispersion

**Figure 1.**
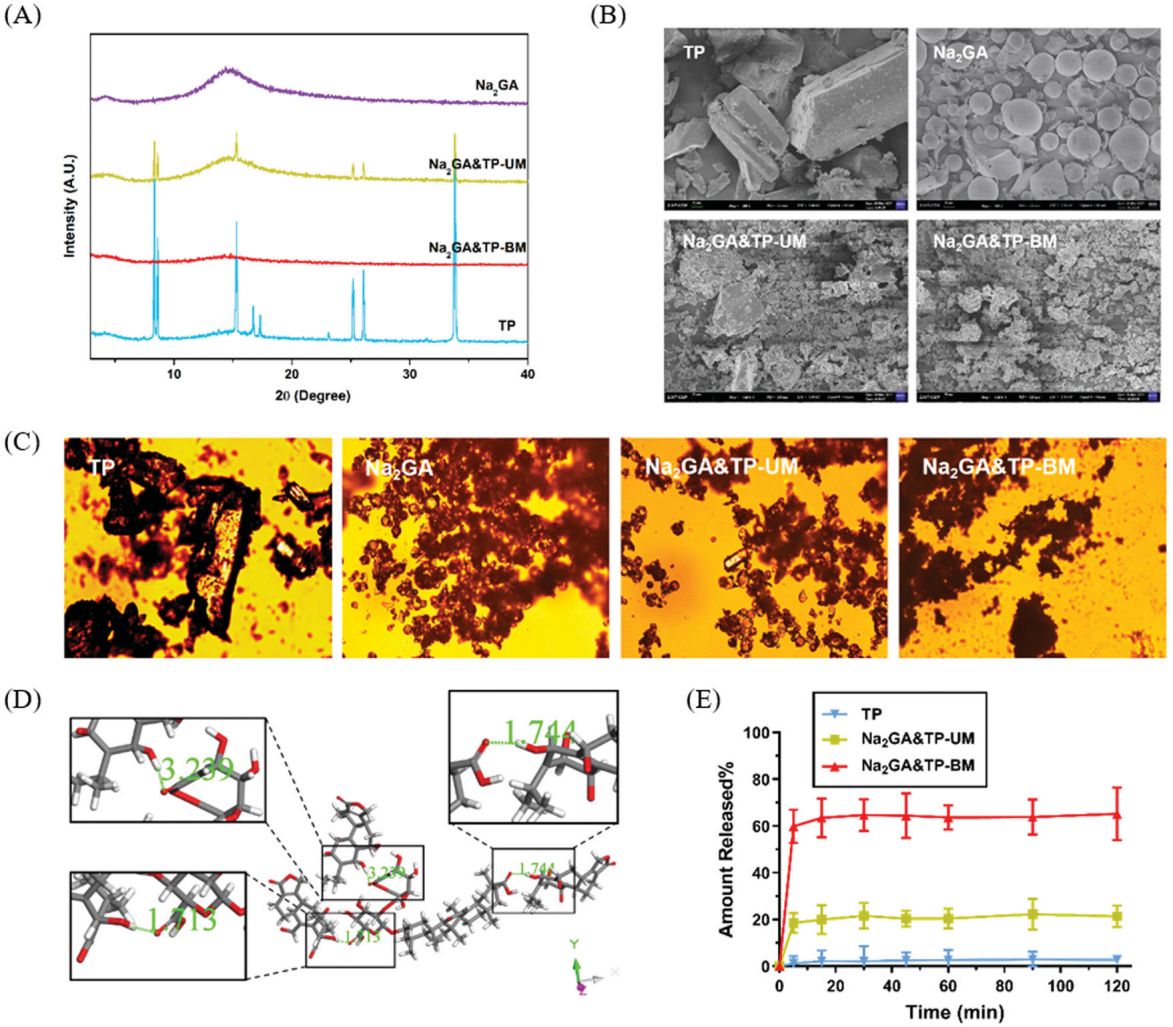
(A) X-ray diffraction spectra of TP, Na_2_GA, Na_2_GA&TP-UM, Na_2_GA&TP-BM. (B) The electron micrographs of TP, Na_2_GA, Na_2_GA&TP-UM, Na_2_GA&TP-BM (the scale bars were 10 μm). (C) PLM images of TP, Na_2_GA, Na_2_GA&TP-UM, Na_2_GA&TP-BM, the magnification was 10×. (D) Hydrogen bond microstructure. (E) *In vitro* release profiles of TP, Na_2_GA&TP-UM, and Na_2_GA&TP-BM (*n* = 3).

Through HPLC detection and analysis, the solubility of the pure TP was 0.1976 mg/mL. Meanwhile, the solubility of 1/10 SD, 1/100 SD (Na_2_GA&TP-BM), and 1/200 SD were 1.0452, 2.3193, and 1.3605 mg/mL. Compared with the solubility of the pure TP, 1/100 SD (Na_2_GA&TP-BM) increased 11.74 times, which was higher than 1/10 SD and 1/200 SD. Therefore, Na_2_GA&TP-BM was chosen as candidate to study the subsequent experiments. The XRD thermograms of Na_2_GA, TP, Na_2_GA&TP-UM, and Na_2_GA&TP-BM are shown in Figure 1(A). The characteristic peak 2*θ* values of TP were detected to be 8.34, 8.62, 15.29, 16.71, 17.30, 25.14, 26,08, and 33.80, indicating its crystalline form. To be noticed that, the physical mixture product Na_2_GA&TP-UM also showed the characteristic peak of TP but decreased significantly, indicating that it is still formed as crystal. However, in the diffraction spectrum of Na_2_GA&TP-BM, the characteristic crystallization peak of TP disappeared completely, identifying that TP had been uniformly distributed in Na_2_GA and formed an amorphous sample by physical ball milling.

PLM and SEM further testified this result. The micrographs of Na_2_GA, TP, Na_2_GA&TP-UM, and Na_2_GA&TP-BM obtained from polarized light microscopy are shown in Figure 1(C). Material in crystal state has obvious birefringence under polarized light microscope. From the images of TP, Na_2_GA&TP-UM, it could be found that there was a mass of crystalline materials, while the birefringence disappeared in Na_2_GA&TP-BM. The electron micrographs of samples were shown in Figure 1(B). It could be visually seen that TP was block solid and the Na_2_GA was in a spherical state. After 30-min grinding, the intact morphology of the TP and Na_2_GA particles was destroyed, forming fine and irregularly shaped particles. Grinding made the solid particles more uniform, which increased the surface area of the solid particles and got better wettability and dispersibility (Descamps & Willart, 2016).

Furthermore, to understand the interaction between TP and GA in water, the hydrogen bonding sites between TP and GA molecules were investigated in detail by the Dmol3 module in the Materials Studio software. TP had fortissimo hydrophobicity and the added TP could promote GA micelle formations, which was a usual observation for the surfactant system mixed with a hydrophobic compound (X Wang & Gao, 2018). the aryl ring of TP had high tendency to hydrophobically interact with the convex hydrophobic surface of GA (Malik, 2016), this may be one of the reasons for the formation of micelles. As shown in Figure 1(D), TP and GA molecules can interact to form three hydrogen bonds, and the hydrogen bond lengths were 1.713, 1.744, and 3.239, respectively. We could speculate that there was hydrogen bonding between the carbonyl and hydroxyl group of TP and the carboxylate group of GA at the suitable arrangement, and the hydrogen bonding between TP and GA was significantly involved in primary micelles (X Wang et al., 2021). Overall, not only the hydrophobic force but also the hydrogen bonding was involved in the binding of TP with Na2GA, and our speculation may need further investigation.

Dissolution profiles of TP, Na_2_GA&TP-UM and Na_2_GA&TP-BM are shown in Figure 1(E). Compared with TP and Na_2_GA&TP-UM, Na_2_GA&TP-BM exhibited better dissolution properties. The cumulative amount of TP dissolved after 5 minutes was 2.4%, 20.7%, and 63.6% for TP, Na_2_GA&TP-UM, and Na_2_GA&TP-BM, respectively. We speculated that the drug was encapsulated in a hydrophilic carrier and formed as the amorphous sample which had better wettability and dispersibility leading to more excellent properties. Na_2_GA&TP-BM form micelles in water, resulting in excellent solubility. While Na_2_GA&TP-UM was not uniformly dispersed in Na_2_GA, therefore only part of TP dissolves rapidly.

### Properties of micelles in water solution

**Figure 2.**
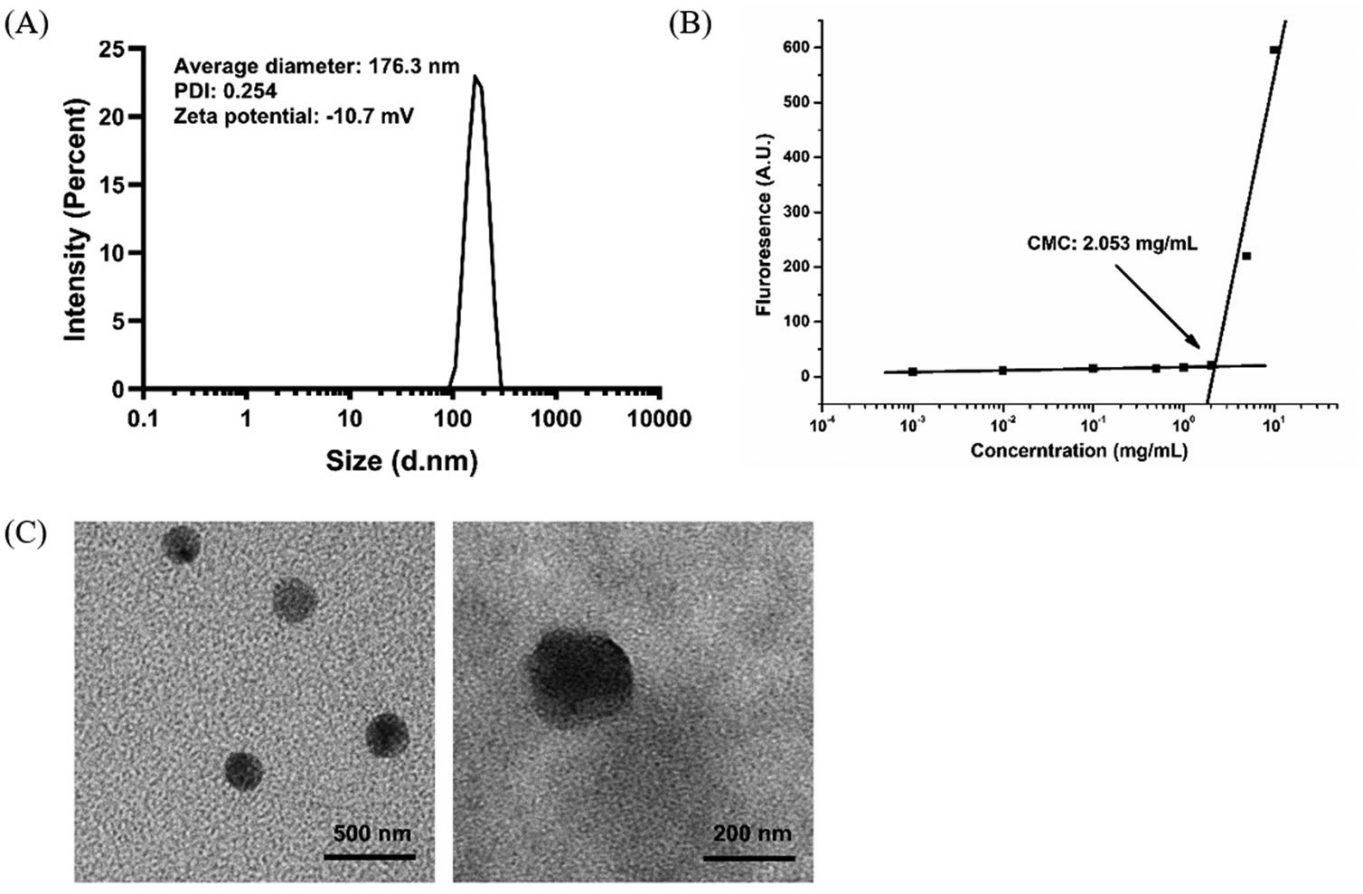
The zeta potential, size, CMC, and surface morphology of Na_2_GA&TP-BM. (A) Dynamic light scattering size measurement of Na_2_GA&TP-BM micelles. (B) The CMC value of Na_2_GA&TP-BM. (C) Transmission electron micrograph (TEM) of Na_2_GA&TP-BM micelles, the scale bars from left to right were 500 nm, 200 nm.

When Na_2_GA&TP-BM dissolved in water, Na_2_GA encapsulated TP to form micelles and the obtained micelle solution was transparent. Figure 2 shows the morphology, ζ-potential, and particle size distribution of Na_2_GA&TP-BM. Under TEM, Na_2_GA&TP-BM was cone-shaped. The particle size of the micelle measured by DLS was 176.3 nm, and PDI was 0.254. Moreover, the ζ-potential of the micelle was −10.7 mV. The CMC was 2.053 mg/mL that analyzed by Origin 9.0 as shown in Figure 2(B). It is reported that most of the average particle sizes which between 100 and 200 nm were considered to be an appropriate size to evade filtration in reticuloendothelial system (RES) organs and were more easily absorbed by the tumor (D Liu et al., 1992; Li & Huang, 2008; Q Sun et al., 2017). Meanwhile, the neutral surface charge of particles (zeta potential ± 10 mV) was proved to prolonged blood circulation and specifically accumulate at the tumor site.

### *In vitro* cellular cytotoxicity

**Figure 3.**
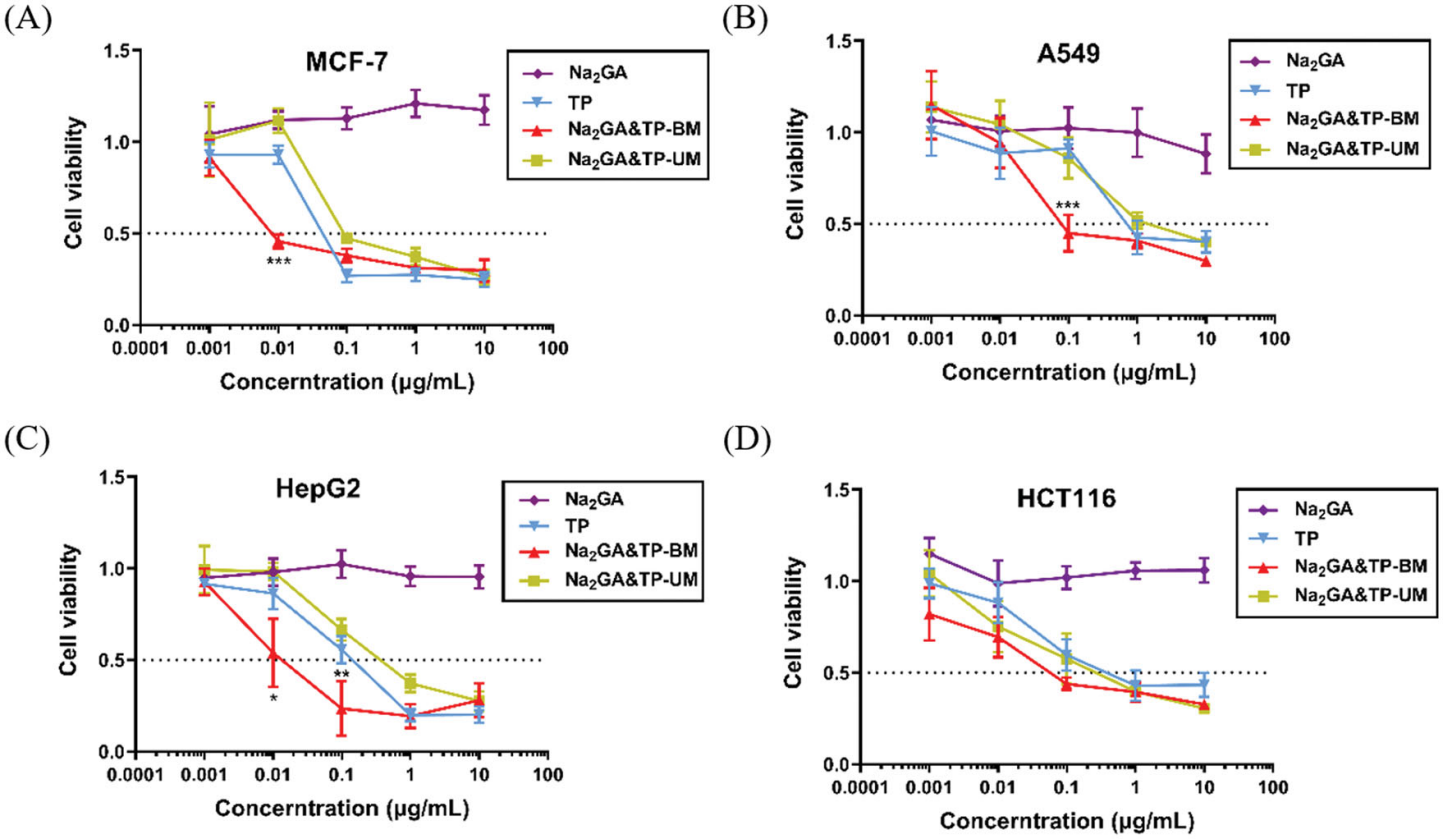
*In vitro* cytotoxicity of TP, Na_2_GA, Na_2_GA&TP-UM, Na_2_GA&TP-BM on MCF-7 cells, A549 cells, HepG2 cells, and HCT116, respectively (*n* = 5). *p <0 .05, **p < 0.01, and ***p <0 .001.

The cytotoxicity of Na_2_GA, free TP, Na_2_GA&TP-UM, and Na_2_GA&TP-BM was tested in several types of cancer cells like MCF-7, A549, HepG2, and HCT116 cells. It showed that free TP, Na_2_GA&TP-UM, and Na_2_GA&TP-BM had the significant inhibition ability in all four cancer cell lines, and the cytotoxicity rose gradually with the increase of the concentration (Figure 3). The half maximal inhibitory concentrations (IC_50_) of TP, Na_2_GA&TP-UM, and Na_2_GA&TP-BM are shown in Table 1. In particular, at a concentration of 0.01 μg/mL, the cytotoxicity of Na_2_GA&TP-BM on MCF-7 and HepG2 cells was statistically significant compared with the other two dosage forms (*p* < .001). The cytotoxicity of Na_2_GA&TP-BM on A549 also had a significant difference at 0.1 μg/mL (*p* < .001). In addition, the survival rate of Na_2_GA group in all four cell lines was above 90%, indicating that Na_2_GA itself possessed no cytotoxicity in all tested cell lines in the concentration range of 0.001–10 µg/mL (Figure 3). Therefore, Na_2_GA&TP-BM enhanced the cytotoxic ability of TP due to the TP’s better absorption on cells. These results confirmed that Na_2_GA&TP-BM was a potential nano-delivery system for cancer treatment.

**Table 1.** The half maximal inhibitory concentrations (IC_50_) of TP, Na_2_GA&TP-UM, and Na_2_GA&TP-BM in MCF-7, HCT116, HepG2 and A549 cells.

Cell lines	IC_50_(μg/mL)		
	TP	Na_2_GA&TP-UM	Na_2_GA&TP-BM
MCF-7	0.076	0.063	0.021
HCT116	0.818	0.483	0.096
HepG2	0.099	0.123	0.006
A549	1.972	2.006	0.297

### Cellular internalization and induction of apoptosis

**Figure 4.**
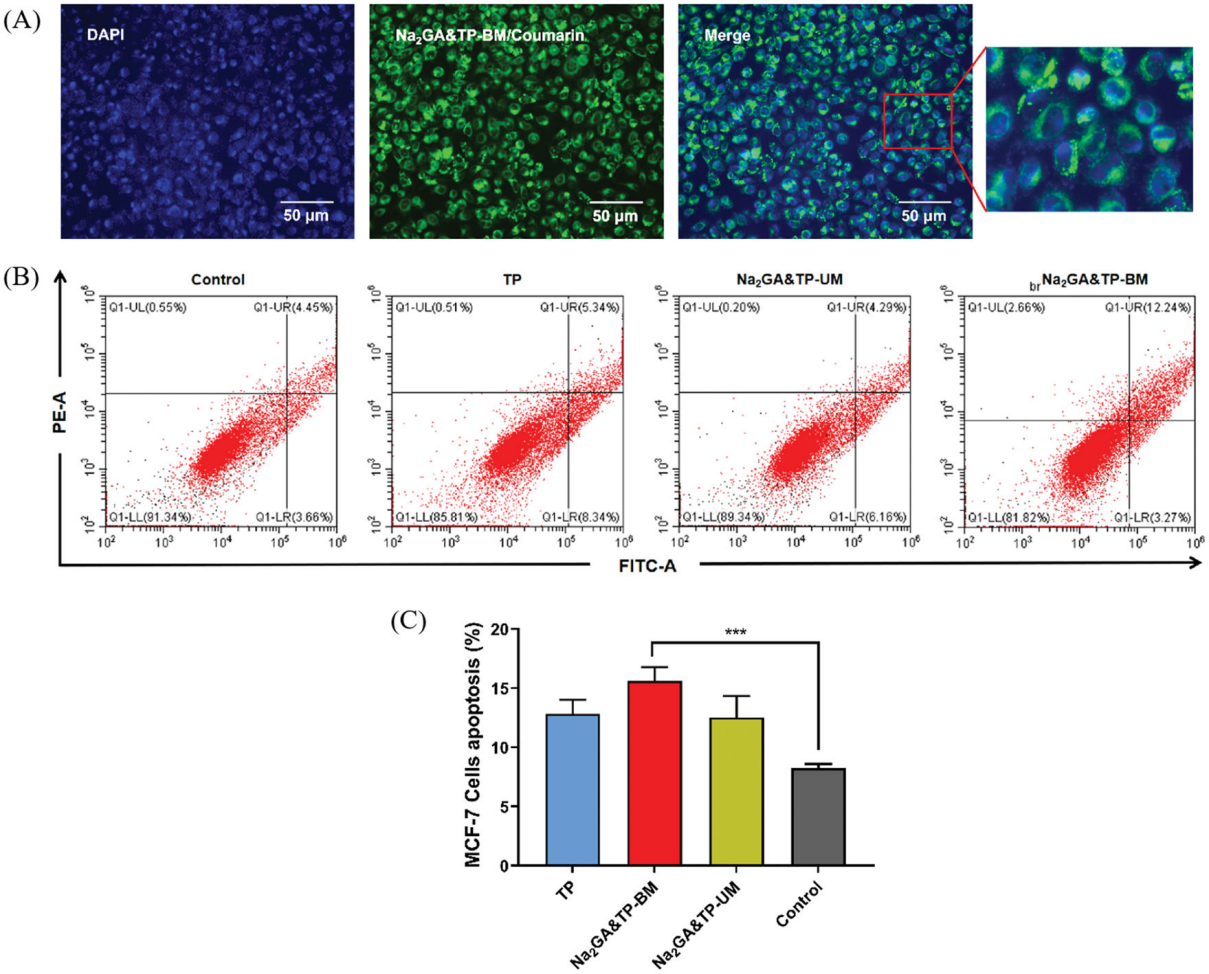
(A) Cell uptake of Na_2_GA&TP-BM/coumarin-6. Nucleus was stained with DAPI. Images were taken from the DAPI channel (blue), Na_2_GA/CA-BM/C6 channel (green), and the overlapped image. (B) Flow cytometric analysis of AnnexinV-FITC/PI stained MCF-37 cells treated with TP, Na_2_GA&TP-UM, Na_2_GA&TP-BM. (C) The apoptotic proportion of MCF-7 cells treated with TP, Na_2_GA&TP-UM, and Na_2_GA&TP-BM. *p <0 .05, **p <0 .01, and ***p <0 .001.

The colocalization and internalization by MCF-7 cells of coumarin-6-loaded Na_2_GA&TP-BM (NPs/C6) was confirmed by CLSM. As shown in Figure 4(A), the fluorescence from NPs/C6 (green) was observed in the cytoplasm after 4 h of incubation at 37 °C, indicating that NPs/C6 could be rapidly taken up by cells and remained in MCF-7 cells.

Subsequently, we examined the apoptosis of MCF-7 cells induced by Na_2_GA&TP-BM. Flow cytometry results showed a significant increase in apoptotic MCF-7 cells with Na_2_GA&TP-BM treatment (Figure 4(B)). The proportion of apoptotic cells (%) was comprised of late apoptotic (district of the upper right) and early apoptotic cells (district of the bottom right). The apoptotic proportion of MCF-7 cells treated with TP, Na_2_GA&TP-UM, and Na_2_GA&TP-BM, reached 12.8%, 12.5% and 15.6%, respectively (Figure 4(C)). Obviously, compared with untreated cells (apoptosis rate: 9.3%), the MCF-7 cells in three test groups showed different degrees of apoptosis, and Na_2_GA&TP-BM groups have the strongest effect which was consistent with cytotoxicity’s conclusion.

### Inhibition growth of 3D tumor spheroids

**Figure 5.**
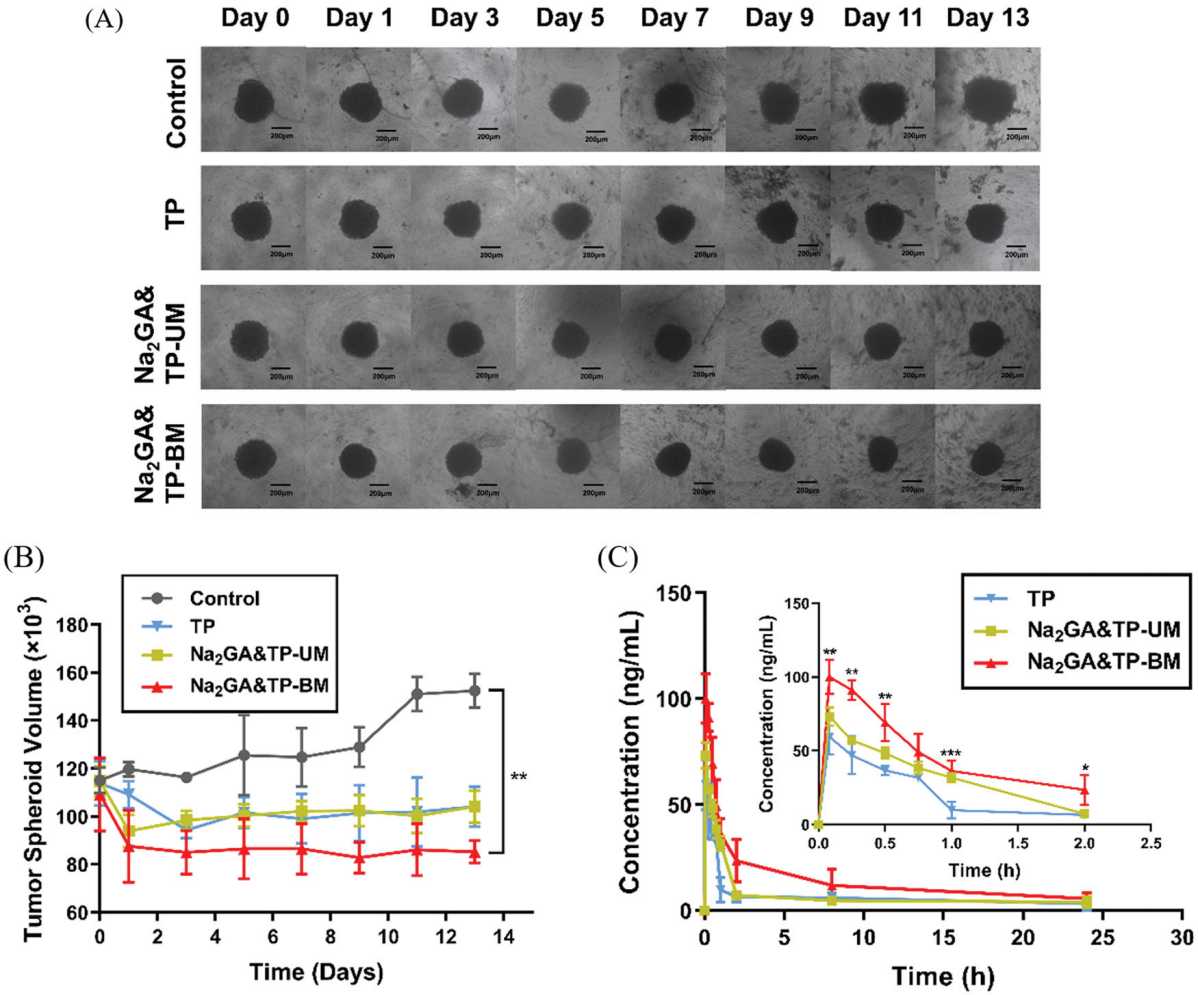
(A) Image-based quantification of the relative average diameter of untreated, TP, Na_2_GA&TP-UM, and Na_2_GA&TP-BM treatment group cells spheroids, The image of multicellular tumor spheroids. (B) Data represent the volume of *n* ≥ 20 cell spheroid. (C) Concentration of TP in rat plasma after intragastric administration of TP, Na_2_GA&TP-UM, and Na_2_GA&TP-BM to rats at the dose of 0.72 mg/kg (*n* = 5), statistical significance compared to TP. *p <0 .05, **p < 0.01, and ***p <0 .001.

As shown in Figure 5(A), MCF-7-tumor spheroids of the control group grew rapidly and gradually became compact when treated with the cell culture medium. The relative diameter of multicellular tumor spheroids in the control group increased by about 32.5% after 13 days’ inoculation. At the same time, pure TP, Na_2_GA&TP-UM, and Na_2_GA&TP-BM groups showed different degrees of decline and decreased by about 8.5%, 10.1%, and 20.9%, respectively, indicating that all TP dosage forms inhibited spheroid cell proliferation to some extent (Figure 5(B)). After 5–7 days’ treatment, the volume of all TP dosage forms groups had obviously shrunk, with some cell detachment from the tumor spheroids. On day 13, Na_2_GA&TP-BM groups penetrated more deeply and distributed more extensively, which was also consistent with the results of *in vitro* cell apoptosis described earlier.

### Pharmacokinetic evaluation

The results of pharmacokinetic studies are listed in Figure 5(C) and Table 2. As shown in Figure 5(C), it could be clearly seen that the bioavailability of Na_2_GA&TP-BM was better than pure TP. After intragastric administration, TP of the three dosage forms distributed rapidly throughout the whole body and reached a peak quickly. In addition, the free TP was cleared faster from blood than Na_2_GA&TP-BM. As shown in Table 2, the bioavailability of the SD rats treated with pure TP, Na_2_GA&TP-UM, and Na_2_GA&TP-BM was 107, 174, and 257 µg/L*h, respectively, and Na_2_GA&TP-BM increased by about 2.5-fold compared with TP. Na_2_GA&TP-BM had a better blood circulation in the body which was also verified by the above theory of the proper particle size of the nanomicelles *in vivo*.

**Table 2.** The pharmacokinetic parameters of pure TP, Na_2_GA&TP-UM and Na_2_GA&TP-BM.

Samples	*C*_max_ (μg/L)	*T*_max_ (h)	*T*_1/2_ (h)	AUC_0→t_ (μg/L*h)	AUC_0→∞_ (μg/L*h)
TP	59.43275	0.083	2.937	81.758	107.216
Na_2_GA&TP-UM	73.1525	0.083	2.493	174.077	174.28
Na_2_GA&TP-BM	100.0615	0.083	3.213	202.053	257.273

### *In vivo* antitumor efficacy

**Figure 6.**
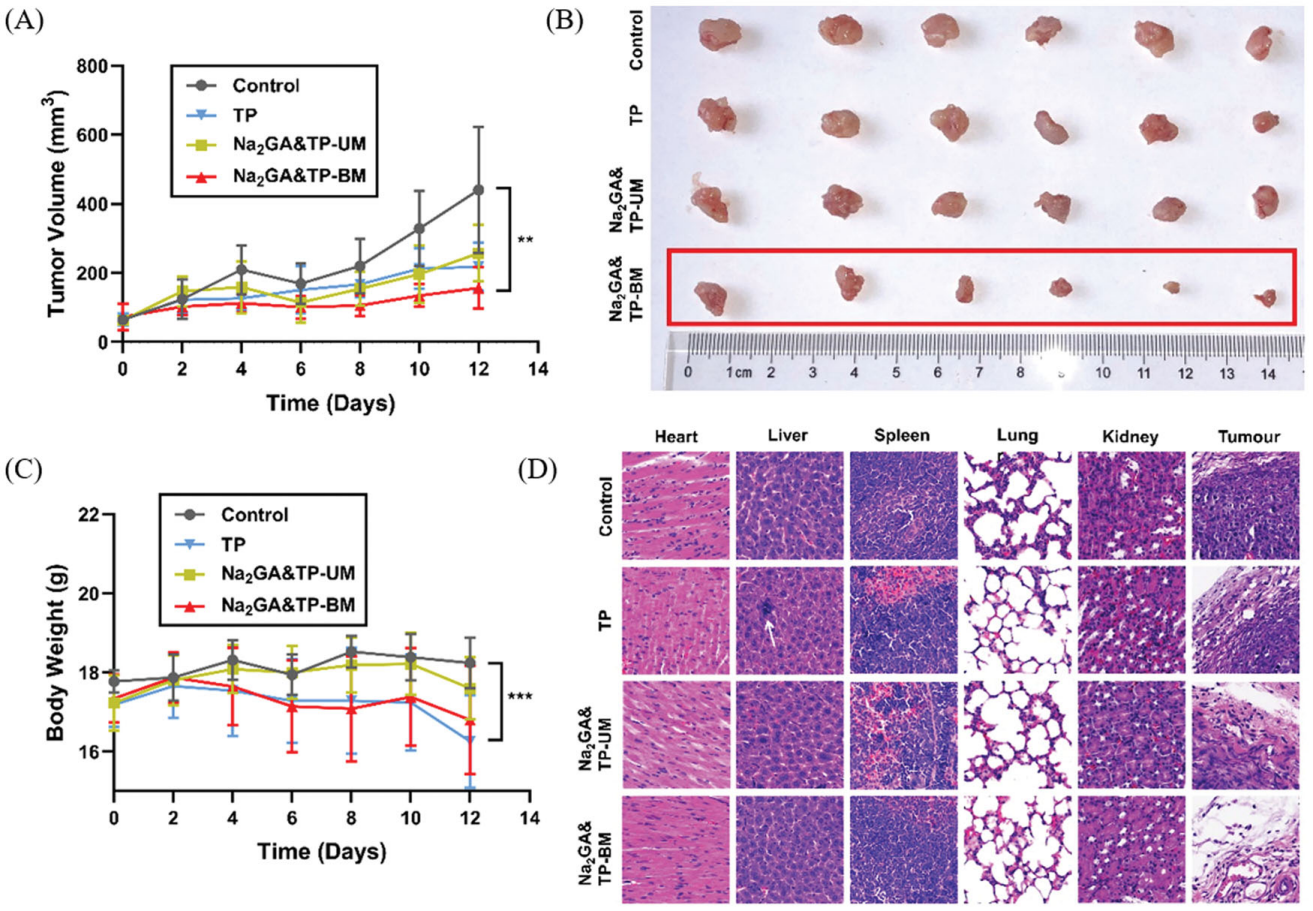
*In vivo* antitumor effect tested in HepG2-bearing mice (*n* = 7) and the pathological sections of major organs and tumors. (A) The change curve of tumor volume throughout the treatment. (B) Solid tumor photograph of control, TP, Na_2_GA&TP-UM, and Na_2_GA&TP-BM treatment group. (C) The body weight curve of mice. (D) H&E staining of major organs and tumor tissue sections with envelop layer. *p <0 .05, **p <0 .01, and ***p < 0.001.

*In vivo* antitumor activity was carried out in nude mice bearing HepG2 tumors. It was demonstrated that the tumor volume of Na_2_GA&TP-BM group remained roughly unchanged with time dependently. Representative results shown in Figure 6(A,B), compared with the control group, Na_2_GA&TP-BM showed better tumor inhibition ability throughout the treatment, nearly 64.48% tumor inhibitory rate. Meanwhile, after 12 days’ treatment, there was no significant difference in tumor volume between TP, Na_2_GA&TP-UM group, and control group (*p* = .200, *p* = .236 compared with the control group).

Next, the pathological sections of main tissues were made to observe the apoptosis of tumor cells and the change of normal organs after treatments. As shown in Figure 6(D), it could be found that the cells of control group were dense and had abundant vascular tissue in tumor tissue. In comparison, especially in the Na_2_GA&TP-BM group, most of the cells with enlarged sizes and a large amount of excessive vacuolization was observed – most tumor cells were apoptotic, indicating that Na_2_GA&TP-BM had the most cytotoxicity. The result was consistent with the above tumor inhibition data. On the whole, the antitumor effect of Na_2_GA&TP-BM was the most prominent of the three formulations.

### *In vivo* safety evaluation

In addition to treatment efficacy, toxicity is another critical parameter of an excellent delivery vehicle for further use. For safety purpose, we evaluated the systematic toxicity of Na_2_GA&TP-BM in healthy BALB/c mice after intragastric administration every other day for 2 weeks. As shown in Figure 6(C), compared with the control group, the Na_2_GA&TP-BM group has no significant difference in body weight during the study period. Meanwhile, TP exhibited potent toxicity. Therefore, it could be seen that Na_2_GA&TP-BM significantly reduced the systemic toxicity of TP drugs.

The safety of TP, Na_2_GA&TP-UM, and Na_2_GA&TP-BM in the main organs was further evaluated by histopathological examination. In Figure 6(D), no obvious organ lesions (including heart, liver, spleen, lung and kidney) were observed in the Na_2_GA&TP-UM group and Na_2_GA&TP-BM group, compared with the control group. However, the liver of mice treated with TP exhibited certain pathological changes.

The results of biochemistry parameters further testified it. As it could be seen in Table 3, the level of plasma ALT in the TP group increased, indicating that pure TP induced liver injury in mice (Yu et al., 2021). Furthermore, The blood indexes of Na_2_GA&TP-UM and Na_2_GA&TP-BM group, included liver functional markers (ALT, AST), kidney functional markers (BUN, CR), myocardial enzyme spectrum (CK, LDH), indicated no significant difference compared with the control group. The anti-inflammatory effects of Na_2_GA may play a role. All of these results showed that multiple dosage of Na_2_GA&TP-BM did not cause acute toxicity to the hematological system and major organs in mice.

**Table 3.** Biochemistry parameters for TP, Na_2_GA&TP-UM, and Na_2_GA&TP-BM-treated nude mice.

Samples	ALT (U/L)	AST (U/L)	BUN (mmol/L)	CR (μmol/L)	LDH (U/L)	CK (U/L)	CK-MB (U/L)
Control	24.33 ± 4.19	189.33 ± 4.03	5.94 ± 0.37	5.67 ± 1.70	3246.33 ± 88.04	1080.67 ± 140.33	1242.97 ± 68.60
TP	42.33 ± 5.73	194.00 ± 9.09	8.54 ± 0.83	4.67 ± 0.47	3302.00 ± 251.02	1098.33 ± 158.68	1272.07 ± 90.61
Na_2_GA&TP-UM	18.67 ± 2.62	193.67 ± 26.41	7.34 ± 0.41	6.33 ± 4.50	3234.00 ± 147.62	892.33 ± 191.95	1022.50 ± 132.07
Na_2_GA&TP-BM	24.67 ± 4.99*	197.33 ± 14.82	11.38 ± 1.37	11.33 ± 4.19	3275.00 ± 148.12	999.67 ± 244.89	1327.07 ± 292.10

There were significant differences in ALT between TP group and Na2GA&TP-BM group. *p <0 .05, **p <0 .01, and ***p < 0.001.

## Conclusions

In summary, Na_2_GA&TP-BM was successfully developed by mechanical ball milling, which overcame the clinical defects of TP in this study. Compared with pure TP and Na_2_GA&TP-UM, the performance of Na_2_GA&TP-BM improved through ball milling, such as from crystalline state to amorphous solid dispersion, suitable nano micelle size and surface potential, and increased solubility. These changes had a significant improvement of pharmacokinetic behavior in mice, which increased about 2.5-fold in oral bioavailability and was able to significantly extend the blood circulation time of the antitumor drug. Moreover, MTT assay and *in vivo* anti-tumor study showed that Na_2_GA&TP-BM displayed more potent cytotoxicity to tumor cells. Preliminary *in vivo* safety studies in mice showed Na_2_GA&TP-BM was safe to oral with minimal or no toxicity to major organs. Collectively, our results showed that Na_2_GA&TP-BM was an environment-friendly and effective anti-tumor preparation of the TP formulation by mechanical ball milling, which warranted further investigation.

## References

[CIT0001] Bahri M, Kazemian H, Rohani S. (2016). Mechanochemical synthesis of CPM-5: a Green method. J Chem Eng Technol 40:88–93.

[CIT0002] Bernela M, Ahuja M, Thakur R. (2016). Enhancement of anti-inflammatory activity of glycyrrhizic acid by encapsulation in chitosan-katira gum nanoparticles. Eur J Pharm Biopharm 105:141–7. 27287555 10.1016/j.ejpb.2016.06.003

[CIT0003] Boldyrev VV. (2005). Mechanochemical modification and synthesis of drugs. J ChemInform 36:5117–20.

[CIT0004] Chen H, Chang X, Weng T, et al. (2004). A study of microemulsion systems for transdermal delivery of triptolide. J Control Release 98:427–36. 15312998 10.1016/j.jconrel.2004.06.001

[CIT0005] Chou CC, Yang JS, Lu HF, et al. (2010). Quercetin-mediated cell cycle arrest and apoptosis involving activation of a caspase cascade through the mitochondrial pathway in human breast cancer MCF-7 cells. Arch Pharm Res 33:1181–91. 20803121 10.1007/s12272-010-0808-y

[CIT0006] Dasa SSK, Suzuki R, Gutknecht M, et al. (2015). Development of target-specific liposomes for delivering small molecule drugs after reperfused myocardial infarction. J Control Release 220:556–67. 26122651 10.1016/j.jconrel.2015.06.017 PMC6113056

[CIT0007] Deng QD, Lei XP, Zhong YH, et al. (2021). Triptolide suppresses the growth and metastasis of non-small cell lung cancer by inhibiting β-catenin-mediated epithelial-mesenchymal transition. Acta Pharmacol Sin 42:1486–97. 33893396 10.1038/s41401-021-00657-w PMC8379262

[CIT0008] Descamps M, Willart JF. (2016). Perspectives on the amorphisation/milling relationship in pharmaceutical materials. Adv Drug Deliv Rev 100:51–66. 26826439 10.1016/j.addr.2016.01.011

[CIT0009] Geszke-Moritz M, Moritz M. (2016). Solid lipid nanoparticles as attractive drug vehicles: composition, properties and therapeutic strategies. Mater Sci Eng C Mater Biol Appl 68:982–94. 27524099 10.1016/j.msec.2016.05.119

[CIT0010] Gu C, Liu F, Luo X, et al. (2016). Triptolide reduces the required dose of tacrolimus by attenuating inflammation, enhancing immunosuppression, and increasing donor chimerism in a heterotopic hindlimb transplantation model. Plast Reconstr Surg 138:1243–53. 27879593 10.1097/PRS.0000000000002770

[CIT0011] Jiang W, Chen M, Xiao C, et al. (2019). Triptolide suppresses growth of breast cancer by targeting HMGB1 in vitro and in vivo. Biol Pharm Bull 42:892–9. 30956264 10.1248/bpb.b18-00818

[CIT0012] Li SD, Huang L. (2008). Pharmacokinetics and biodistribution of nanoparticles. Mol Pharm 5:496–504. 18611037 10.1021/mp800049w

[CIT0013] Ling D, Xia H, Park W, et al. (2014). pH-sensitive nanoformulated triptolide as a targeted therapeutic strategy for hepatocellular carcinoma. ACS Nano 8:8027–39. 25093274 10.1021/nn502074x

[CIT0014] Liu D, Mori A, Huang L. (1992). Role of liposome size and RES blockade in controlling biodistribution and tumor uptake of GM1-containing liposomes. Biochim Biophys Acta 1104:95–101. 1550858 10.1016/0005-2736(92)90136-a

[CIT0015] Liu L, Xiong X, Shen M, et al. (2018). Co-delivery of triptolide and curcumin for ovarian cancer targeting therapy via mPEG-DPPE/CaP nanoparticle. J Biomed Nanotechnol 14:1761–72. 30041722 10.1166/jbn.2018.2633

[CIT0016] Malik NA. (2016). Solubilization and interaction studies of bile salts with surfactants and drugs: a review. Appl Biochem Biotechnol 179:179–201. 26781714 10.1007/s12010-016-1987-x

[CIT0017] Monteiro N, Martins A, Reis RL, et al. (2014). Liposomes in tissue engineering and regenerative medicine. J R Soc Interface 11:20140459. 25401172 10.1098/rsif.2014.0459 PMC4223894

[CIT0018] Pompei R, Laconi S, Ingianni A. (2009). Antiviral properties of glycyrrhizic acid and its semisynthetic derivatives. Mini Rev Med Chem 9:996–1001. 19601894 10.2174/138955709788681636

[CIT0019] Ren Q, Li M, Deng Y, et al. (2021). Triptolide delivery: nanotechnology-based carrier systems to enhance efficacy and limit toxicity. Pharmacol Res 165:105377. 33484817 10.1016/j.phrs.2020.105377

[CIT0020] Ridolfo R, Ede BC, Diamanti P, et al. (2018). Biodegradable, drug-loaded nanovectors via direct hydration as a new platform for cancer therapeutics. Small (Weinheim an Der Bergstrasse, Germany) 14:e1703774. 29999236 10.1002/smll.201703774

[CIT0021] Su X, Wu L, Hu M, et al. (2017). Glycyrrhizic acid: a promising carrier material for anticancer therapy. Biomed Pharmacother 95:670–8. 28886526 10.1016/j.biopha.2017.08.123

[CIT0022] Sun Q, Zhou Z, Qiu N, et al. (2017). Rational design of cancer nanomedicine: nanoproperty integration and synchronization. Adv Mater 29:1606628. 10.1002/adma.201606628 28234430

[CIT0023] Sun X, Zhu D, Cai Y, et al. (2019). One-step mechanochemical preparation and prominent antitumor activity of SN-38 self-micelle solid dispersion. Int J Nanomedicine 14:2115–26. 30988612 10.2147/IJN.S193783 PMC6440449

[CIT0024] Tian L, Zhang Y, Wang Y, et al. (2019). Triptolide reduces proliferation and enhances apoptosis in drug-resistant human oral cancer cells. Int J Clin Exp Pathol 12:1204–13. 31933935 PMC6947074

[CIT0025] Wang X, Gao Y. (2018). Effects of length and unsaturation of the alkyl chain on the hydrophobic binding of curcumin with Tween micelles. Food Chem 246:242–8. 29291845 10.1016/j.foodchem.2017.11.024

[CIT0026] Wang K, Guo C, Zou S, et al. (2018). Synthesis, characterization and in vitro/in vivo evaluation of novel reduction-sensitive hybrid nano-echinus-like nanomedicine. Artif Cells Nanomed Biotechnol 46:659–67. 29703084 10.1080/21691401.2018.1466147

[CIT0027] Wang X, Zhang S, Zhao H, et al. (2021). Spectroscopic investigation into the binding of erulic acid with sodium deoxycholate: hydrophobic force versus hydrogen bonding. Langmuir 37:1420–8. 33475381 10.1021/acs.langmuir.0c02880

[CIT0028] Wei G, Li Y, Zhang L, et al. (2018). Synthesis of bentonite-supported Fe(II) and heteropolyacid (HPW) composite through a mechanochemical processing. J Appl Clay Sci 152:342–51.

[CIT0029] Xu L, Qiu Y, Xu H, et al. (2013). Acute and subacute toxicity studies on triptolide and triptolide-loaded polymeric micelles following intravenous administration in rodents. Food Chem Toxicol 57:371–9. 23583804 10.1016/j.fct.2013.03.044

[CIT0030] Xu W, Wen M, Yu J, et al. (2018). Mechanochemical preparation of kaempferol intermolecular complexes for enhancing the solubility and bioavailability. Drug Dev Ind Pharm 44:1924–32. 30035618 10.1080/03639045.2018.1503292

[CIT0031] Xue M, Zhao Y, Li XJ, et al. (2012). Comparison of toxicokinetic and tissue distribution of triptolide-loaded solid lipid nanoparticles vs free triptolide in rats. Eur J Pharm Sci 47:713–7. 22677813 10.1016/j.ejps.2012.05.012

[CIT0032] Yang S, Chen J, Guo Z, et al. (2003). Triptolide inhibits the growth and metastasis of solid tumors. Mol Cancer Ther 2:65–72. 12533674

[CIT0033] Ye J, Li R, Yang Y, et al. (2021). Comparative colloidal stability, antitumor efficacy, and immunosuppressive effect of commercial paclitaxel nanoformulations. J Nanobiotechnol 19:199. 10.1186/s12951-021-00946-w PMC8256566 34225762

[CIT0034] Yu L, Wang Z, Mo Z, et al. (2021). Synergetic delivery of triptolide and Ce6 with light-activatable liposomes for efficient hepatocellular carcinoma therapy. Acta Pharm Sin B 11:2004–15. 34386334 10.1016/j.apsb.2021.02.001 PMC8343191

[CIT0035] Zhang C, Peng F, Liu W, et al. (2014). Nanostructured lipid carriers as a novel oral delivery system for triptolide: induced changes in pharmacokinetics profile associated with reduced toxicity in male rats. IJN 9:1049–63. 24591827 10.2147/IJN.S55144 PMC3934590

[CIT0036] Zhang Q, Polyakov NE, Chistyachenko YS, et al. (2018). Preparation of curcumin self-micelle solid dispersion with enhanced bioavailability and cytotoxic activity by mechanochemistry. Drug Deliv 25:198–209. 29302995 10.1080/10717544.2017.1422298 PMC6058497

[CIT0037] Zhu J, Zheng S, Liu H, et al. (2021). Evaluation of anti-tumor effects of crocin on a novel 3D tissue-engineered tumor model based on sodium alginate/gelatin microbead. Int J Biol Macromol 174:339–51. 33529625 10.1016/j.ijbiomac.2021.01.181

